# Tumor-to-tumor metastasis: an extremely rare combination with renal cell carcinoma as the donor and a pancreatic neuroendocrine tumor as the recipient

**DOI:** 10.1186/s40792-022-01361-5

**Published:** 2022-01-10

**Authors:** Shunryo Minezaki, Takeyuki Misawa, Hiroyuki Tsukayama, Makoto Shibuya, Keita Wada, Keiji Sano, Makoto Mochizuki, Yuko Sasajima, Hiroshi Kondo

**Affiliations:** 1 Department of Surgery, Teikyo University School of Medicine, 2-11-1 Kaga, Itabashi-Ku, Tokyo, 173-8606 Japan; 2 Department of Pathology, Teikyo University Hospital, 2-11-1 Kaga, Itabashi-Ku, Tokyo, 173-8606 Japan; 3 Department of Radiology, Teikyo University School of Medicine, 2-11-1 Kaga, Itabashi-Ku, Tokyo, 173-8606 Japan

**Keywords:** Clear cell renal cell carcinoma, Pancreatic neuroendocrine tumor, Tumor-to-tumor metastasis

## Abstract

**Background:**

Tumor-to-tumor metastasis is a rare phenomenon in which primary tumor cells metastasize hematogenously into another tumor. Herein, we report an extremely rare case of a renal cell carcinoma metastasis into a pancreatic neuroendocrine tumor exhibiting a tumor-to-tumor metastasis. Ours is the third reported case worldwide.

**Case presentation:**

The patient, a 72-year-old male, was referred to our hospital for further examination and treatment due to high levels of prostate-specific antigen. A left renal tumor and pancreatic head tumor were revealed incidentally on screening computed tomography. There were suspected to be a renal cell carcinoma and primary pancreatic neuroendocrine tumor or pancreatic metastasis from the renal cell carcinoma according to preoperative examination. The left nephrectomy and subtotal stomach-preserving pancreaticoduodenectomy were performed because of the pancreatic tumor indicated for operation in either case of diagnosis. Postoperative pathological examination showed a diagnosis of clear cell renal cell carcinoma for the left renal tumor. The pancreatic tumor was diagnosed with clear cell renal cell carcinoma metastasis into the pancreatic neuroendocrine tumor, that is to say tumor-to-tumor metastasis.

**Conclusion:**

In some cases, conservative approach is selected for pancreatic neuroendocrine tumor patients who meet some requirements. However, if such patients exhibit tumor-to-tumor metastasis which combines with renal cell carcinoma and pancreatic neuroendocrine tumor as this case, conservative approach leads to progression of renal cell carcinoma. Therefore, conceiving the possibility of tumor-to-tumor metastasis, it is necessary to carefully choose a treatment plan for pancreatic neuroendocrine tumor patients associated with renal cell carcinoma, not easily choosing conservative approach.

## Background

Both the "seed and soil theory" and "anatomical-mechanical theory" have been indicated to contribute to the mechanism of tumor metastasis [1, 2]. The phenomenon of metastasis is generally known to depend on the interaction of metastatic tumor cells and the microenvironment of the target organ [3]. Tumor-to-tumor metastasis (TTM), in which primary tumor cells metastasize into other tumors, is a rare condition requiring four criteria for diagnosis, and many kinds of tumors associated with TTM have been reported [4]. However, the underlying mechanism for TTM is unclear due to the small number of associated case reports.

Treatment of nonfunctional pancreatic neuroendocrine tumor (PNET) is controversial. Surgical treatment is generally recommended for PNET patients; however, conservative approach is selected for the patients who meet some requirements [5–9]. Herein, we report an extremely rare case of renal cell carcinoma (RCC) that had metastasized into a PNET and thus presenting as TTM.

## Case presentation

**Fig. 1:**
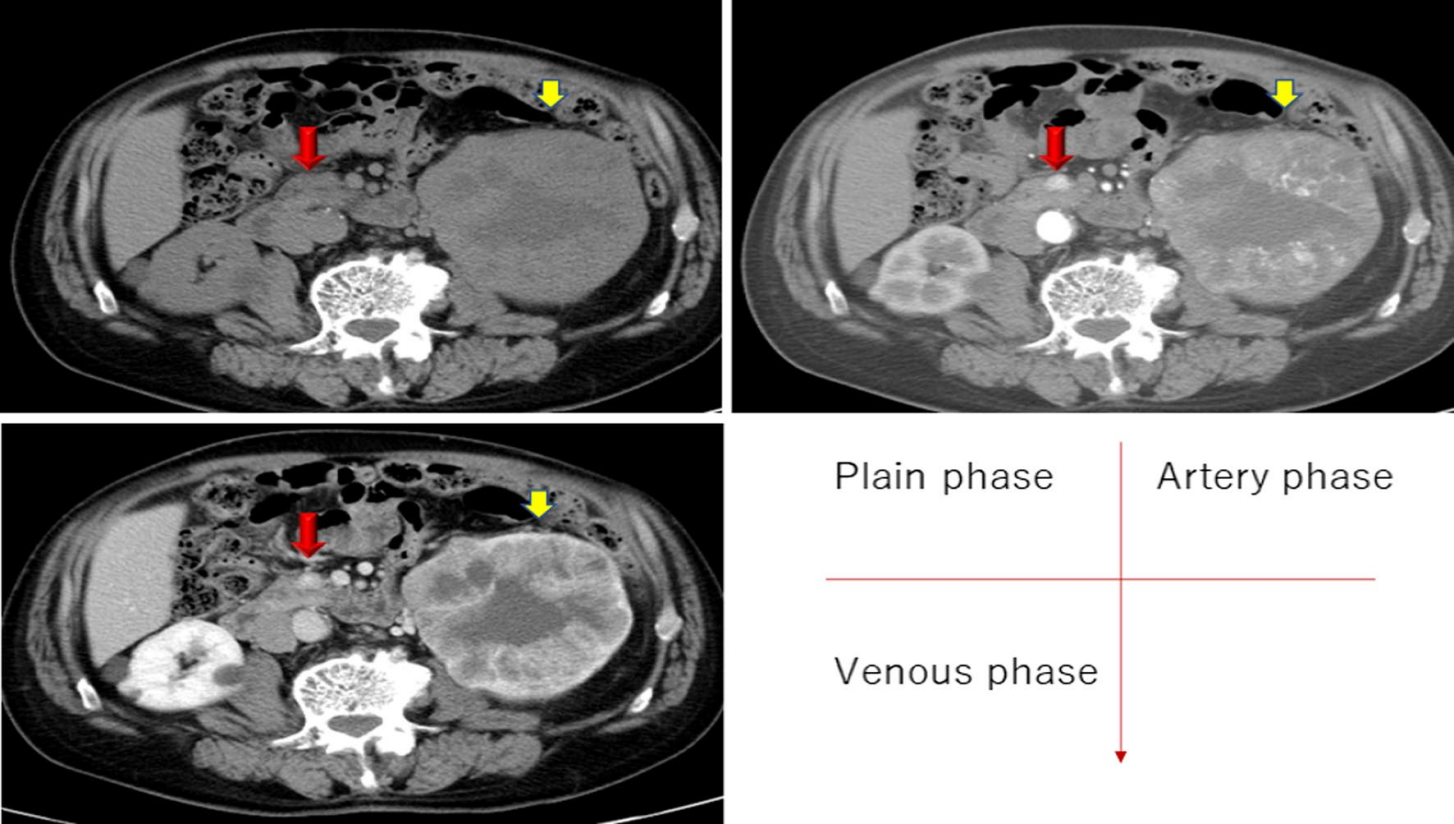
Enhanced CT findings of the RCC and pancreatic head tumor. The renal tumor (yellow arrow) was 10 cm in diameter and demonstrated enhancement in the arterial phase and even more enhancement in the venous phase, but less enhancement than that in the normal right kidney. The tumor was diagnosed as an RCC with necrotic components. The pancreatic head tumor (red arrow), measuring 1 cm in diameter, was well enhanced in both the arterial and venous phases. The tumor was diagnosed as a metastasis from the RCC or a primary PNET

**Fig. 2:**
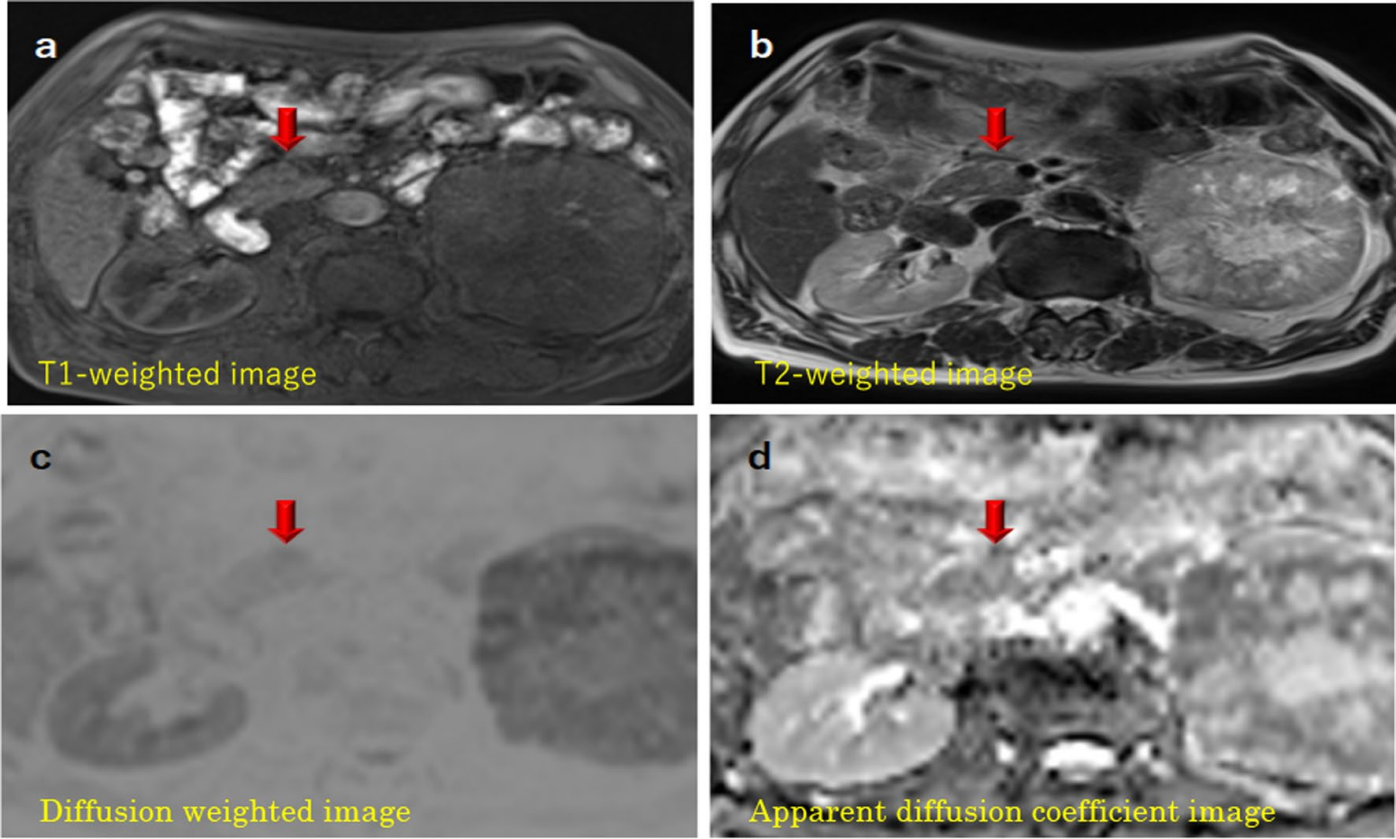
Gd-DTPA-enhanced MRI findings of the pancreatic head tumor. The pancreatic tumor presented with homogenous hypointensity on **a** T1-weighted imaging and **b** T2-weighted imaging. **c** The pancreatic tumor presented with mild hyperintensity on diffusion-weighted imaging. **d** The aforementioned hypointensity was unclear on apparent diffusion coefficient mapping

The patient was a 72-year-old male who was referred to the Department of Urology in our hospital due to a high level of prostate-specific antigen. Blood examination showed RBC 508 * 10^4^/µl, Plt 35.6 * 10^4^/µl, WBC 5700/µl, AST 20 U/L, ALT 16 U/L, LDH 217 U/L, Alp 310 U/L, γ-GTP 24 U/L, Amy 90 U/L, CRP 0.35 mg/dl, CA19-9 6.2 U/ml, CEA 4.1 ng/ml, and PSA 5.025 ng/ml. Enhanced computed tomography showed a sporadic small lung nodule lesion and a renal tumor 10 cm in diameter, which showed enhancement in the arterial phase and even greater enhancement in the venous phase but less than that in the normal right kidney. The renal tumor was diagnosed as an RCC with necrotic components, and the lung lesion was diagnosed as lung metastasis from the RCC. A 1 cm pancreatic head tumor was well enhanced in both the arterial and venous phases. The tumor was diagnosed as metastasis from the RCC or the primary PNET (Fig. 1). Gadolinium-diethylene triamine pentaacetate-enhanced magnetic resonance imaging showed that the pancreatic head tumor presented with homogenous hypointensity on T1-weighted imaging and T2-weighted imaging. Mild hyperintensity was shown on diffusion-weighted imaging, but the hypointensity was unclear on apparent diffusion coefficient mapping. The pancreatic head tumor intensity on T2-weighted imaging was different from that of the neuroendocrine tumor, and the dynamic enhancement findings were consistent with those of the RCC. Therefore, the pancreatic tumor was suspected to be a pancreatic metastasis from the RCC rather than the asymptomatic and nonfunctional PNET (Fig. 2a–d).

The pancreatic tumor was indicated for operation with either diagnosis, and left nephrectomy and subtotal stomach-preserving pancreaticoduodenectomy were performed. Although a grade B pancreatic fistula occurred, it was conservatively improved and the patient was discharged on postoperative day 26. Three years after surgery, the unresected lung metastasis is stable without recurrent disease under nivolumab administration.

**Fig. 3:**
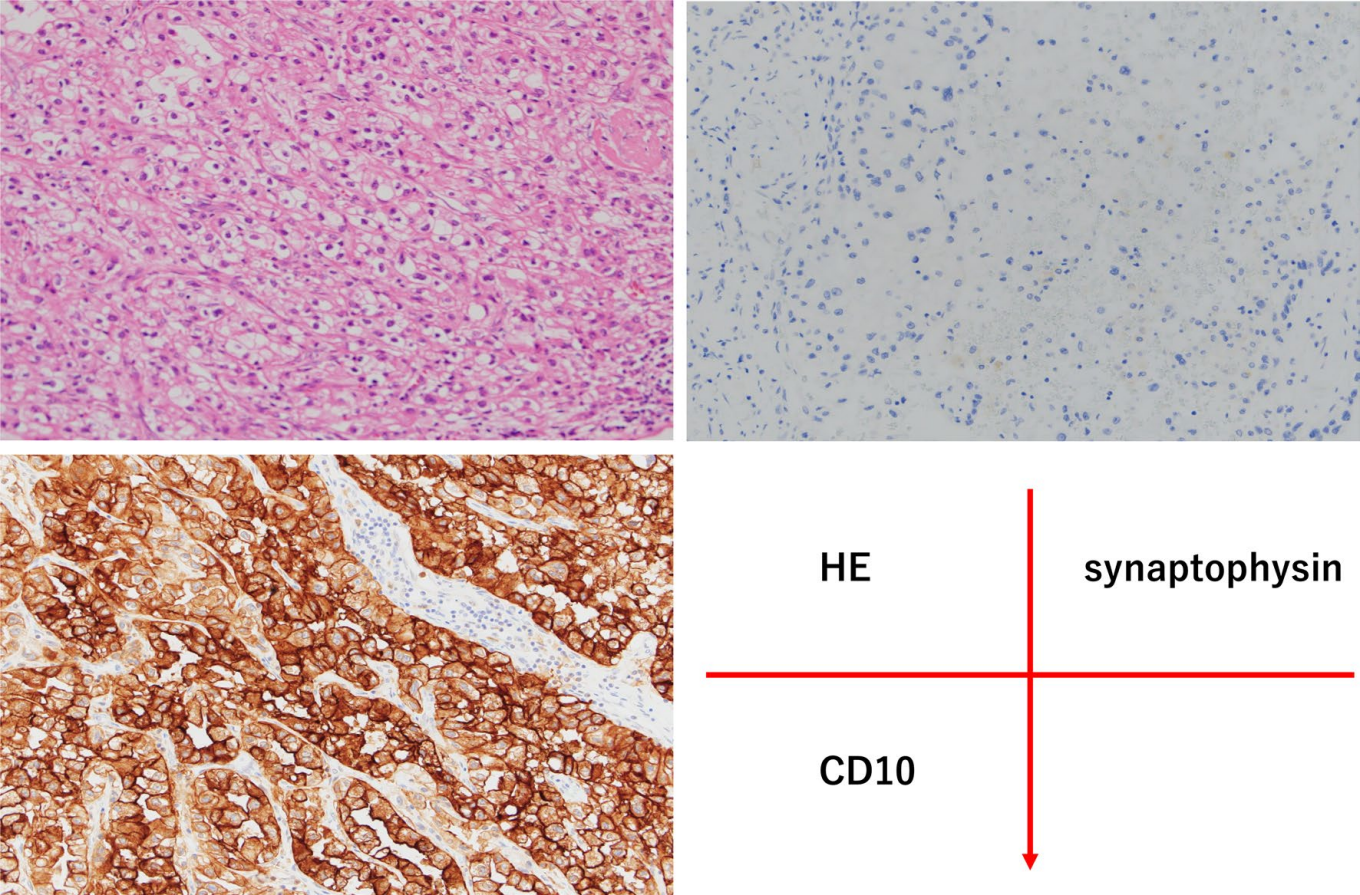
Histopathological findings of the left kidney tumor. Large-sized tumor cells with atypical small nucleus and clear cytoplasm made trabecular nests with intervening thin-walled vascular network with hematoxylin and eosin (HE) stain. These tumor cells were negative for synaptophysin stain and chromogranin A stain, but positive for CD10. We diagnosed this tumor as clear cell RCC

**Fig. 4:**
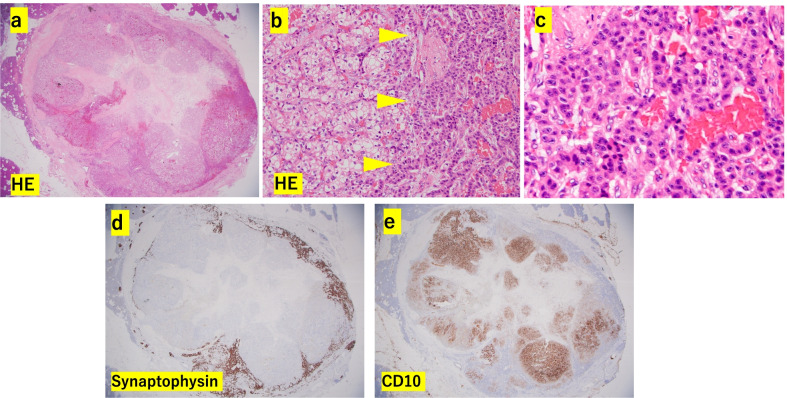
Histopathological findings of the pancreatic tumor which exhibited TTM combined with neuroendocrine tumor and RCC. **a** Overall view of the pancreatic tumor with HE stain. The tumor was composed of two different components which were divided into the central and peripheral areas. **b** Boundary area of the central and peripheral area. Left side area from the yellow arrowhead showed central area in the pancreatic tumor; this area composed the same findings to clear cell RCC of the left kidney tumor. Right side from the yellow arrowhead area showed peripheral area in the pancreatic tumor; this area was considered as neuroendocrine tumor. **c** The peripheral area of the pancreatic tumor with HE stain of a high power field figure. Medium-sized tumor cells with fine granular cytoplasm and round nucleus made ribbon-like regular cord structures. We diagnosed with this area tumor as PNET. **d** Positive for synaptophysin stain in the peripheral area, whereas negative in the central area. **e** Negative for CD10 stain in the peripheral area, whereas positive in the central area

Resected left kidney included a 12 × 10 × 10 cm-sized yellow nodular tumor. Histologically large-sized tumor cells with atypical small nucleus and clear cytoplasm made trabecular nests with intervening thin-walled vascular network. Venous invasion and invasion to perirenal adipose tissue were observed. In immunostaining this tumor was negative for synaptophysin, chromogranin A, CD56, and positive for CD10 (Fig. 3). This kidney tumor was diagnosed for clear cell RCC, Grade 3 (Fuhrman classification), pT3aNX. Resected pancreas tissue included a 1.2 × 1.1 cm-sized well-demarcated nodular tumor. Histologically, the nodule was composed of two different components. In the central area, large-sized tumor cells with atypical small nucleus and clear cytoplasm made trabecular nests with intervening thin-walled vascular network. In immunostaining this tumor was negative for synaptophysin, chromogranin A, CD56, and positive for CD10. These were exactly the same findings to clear cell RCC of the left kidney. In the peripheral area of the nodule, medium-sized tumor cells with fine granular cytoplasm and round nucleus made ribbon-like regular cord structures. Few mitotic figures were identified. In immunostaining, the tumor was positive for synaptophysin, chromogranin A, and negative for CD10 and CD56 (Fig. 4a–e). This peripheral tumor was considered as neuroendocrine tumor G1 originated in pancreas. These findings in this patient were considered that clear cell RCC of the left kidney metastasized into the nodule of the PNET (tumor-to-tumor metastasis).

## Discussion

TTM is a very rare pathology in which one tumor (donor) metastasizes hematogenous into another type of tumor (recipient), thus establishing a secondary tumor [10]. Four criteria have been outlined for a diagnosis of TTM: (1) more than two primary tumors must be present; (2) the host tumor (recipient) must be a true neoplasm; (3) the metastatic tumor (donor) must establish growth inside of the host tumor (recipient) excepting the result of contiguous growth, a collision tumor, or embolization; and (4) the host tumor (recipient) must not be a lymph node associating with leukemia or lymphoma [4]. To date, approximately 150 cases of TTM have been reported in studies worldwide [10]. Meningioma are the most frequent recipient tumors, and breast and lung cancer are the most frequent donors [11]. Our RCC (donor) to PNET (recipient) TTM is the third reported case in the worldwide literature [12, 13].

The cause of TTM is unclear; however, three conditions for the recipient tumor are necessary for the establishment of TTM: (1) it must be hypervascularity, allowing it to be affected by hematogenous metastasis [14]; (2) it must be richly nourished, allowing the growth of donor tumor cells [15]; and (3) it should be characterized by slow growth, providing enough time for the metastatic donor tumor cells to develop [16]. PNETs are generally known to be characterized by hypervascularity and slow growth [17, 18]; therefore, this kind of tumor may present with the necessary conditions to serve as a recipient.

It is generally known that RCC is the most frequent origin of pancreatic metastasis in surgically resected cases [19]. Although the mechanism of pancreatic metastasis from RCCs remains controversial, hematogenous metastasis due to collateral vessels or lymphatic metastasis are thought to contribute. The former suggests that RCC cells metastasize to the pancreas via the formation of collateral venous tracts due to renal vein thrombosis, while the latter indicates that RCC cells metastasize retrogradely of lymph flow following invasion to the retroperitoneal lymph nodes [20]. The collateral venous tract includes a flow into the splenic vein via the subphrenic vein, gastroepiploic vein, or the flowing tract in the duodenal vein from a branch of the retroperitoneal vein [21].

The pancreas is a hypervascular organ with endocrine and exocrine functions, and PNET is a more hypervascular tumor [17]. Therefore, regarding the possibility of hematogenous metastasis in this case, RCC cells were more likely to have greater flow into the PNET than into the normal pancreatic tissue. As a result, it was inferred that metastatic lesions might have formed in the PNET, resulting in the formation of TTM. Of course, as mentioned above, the possibility of lymphatic metastasis cannot be denied, but it was not possible to demonstrate this mechanism given the findings in this case.

In the nineteenth century, Stephen Paget described the organ tropism theory of metastatic tumors [1]. According to this theory, the tumor demonstrates a metastatic tendency to certain organs independent of the anatomy of the blood vessels, the proportion of blood flow, or the number of tumor cells transferred to the target organ [22]. Currently, the metastatic ability of tumor cells is generally known to depend on the interaction of tumor cells and the microenvironment of the target organ. In other words, specific binding between endothelial cells in the vasculature of the target organ and metastatic tumor cells and on the reaction of local growth factors that are secreted in the target organ. This interaction is described as the affinity between metastatic tumor cells and the target organ, also known as “cross-talk” [3]. In fact, the cross-talk between mesothelin, a glycoprotein expressed on the cell membrane, and mucin-16 is thought to play a role in invasion and metastasis in TTM, in which adenocarcinoma, as the donor, metastasizes into meningioma, the recipient [23]. However, despite a PubMed search performed using the terms “pancreatic neuroendocrine tumor” and “renal cell carcinoma”, the literature regarding the molecular markers that play an important role in metastasis and invasion associated with both terms was not reviewed.

A comparison with previous reports revealed that, although many differences were found, PNETs were commonly found to be small tumors of 2 cm or less, and the diagnosis of TTM was difficult by preoperative imaging [12, 13]. PNETs and pancreatic metastases from RCC are both hypervascular tumor; therefore, distinguishing the imaging findings of small PNETs and pancreatic metastases from RCC is difficult. The mortality of pancreatoduodenectomy is 1.3–2.7%; therefore, this surgery is generally known as highly invasive surgery [24, 25]. Therefore, a treatment plan of asymptomatic and nonfunctional PNET patients who meet some requirements, small tumor size or no revelation of metastatic and invasion findings, is selected conservative approach in some cases [5, 6, 9]. However, if a conservative approach was facilely selected for PNET patients with a history of RCC treatment, the patients who exhibited TTM as in this case and previous reports may have missed a treatment chance due to leading RCC progression. Preoperative diagnosis of a combination of PNET and RCC exhibiting TTM is difficult. Therefore, the treatment strategy for patients with PNETs who have previously been treated for RCC should be chosen carefully, taking into account the possibility of TTM.

## Conclusion

This is the third case worldwide in which RCC was found to have metastasized into a PNET, resulting in TTM. Preoperative diagnosis of TTM is difficult; therefore, if the possibility of TTM is not able to conceive, conservative approach may be selected for PNET patients who meet some requirements. Conceiving the possibility of TTM, it is necessary to carefully choose a treatment plan for PNET patients with a history of RCC, not easily choosing conservative approach.

## Data Availability

Data sharing is not applicable to this article.

## Abbreviations

PNET: Pancreatic neuroendocrine tumor
RCC: Renal cell carcinoma
TTM: Tumor-to-tumor metastasis
HE: Hematoxylin and eosin
